# Remote ischemic preconditioning in elderly patients with acute myocardial infarction and transient ischemic attack: a retrospective cohort study

**DOI:** 10.3389/fcvm.2026.1735859

**Published:** 2026-05-29

**Authors:** Yunbo Xie, Hanjun Pei, Lin Liu, Yue Wang

**Affiliations:** 1 Department of Cardiology, The First Affiliated Hospital of Baotou Medical College, Inner Mongolia University of Science and Technology, Baotou, Inner Mongolia, China; 2 Department of Cardiology, Zhangjiakou First Hospital, Zhangjiakou, Hebei, China

**Keywords:** acute myocardial infarction, inflammation, major adverse cardiac and cerebrovascular events, remote ischemic preconditioning, T lymphocytes, transient ischemic attack

## Abstract

**Background:**

Elderly patients who experience acute myocardial infarction (AMI) in temporal proximity to transient ischemic attack (TIA) carry a high inflammatory burden and a disproportionate risk of recurrent cardio-cerebrovascular events. Remote ischemic preconditioning (RIPC) has been hypothesized to provide systemic protection through neurohumoral and immunomodulatory pathways, but clinical data in this dual-organ ischemia phenotype are limited.

**Methods:**

We performed a single-center retrospective cohort study (2018–2023) of patients aged ≥60 years with AMI complicated by neurologist-adjudicated TIA. Patients received guideline-directed care with or without adjunctive RIPC per treating physician judgment. The RIPC protocol consisted of four cycles of 5-min brachial cuff inflation (200 mm Hg or ≥20 mm Hg above systolic pressure) and 5-min deflation, twice daily for 14 days, initiated within 24 h when feasible. The primary endpoint was 12-month major adverse cardiac and cerebrovascular events (MACCE). Time-to-event analyses included Kaplan–Meier estimates and Cox models; the multivariable model adjusted for age, sex, diabetes, left-ventricular ejection fraction (LVEF), log_e interleukin−6 (IL-6), high-sensitivity C-reactive protein (hs-CRP), and ACE-inhibitor therapy.

**Results:**

Of 382 patients screened, 103 were analyzed (RIPC, 48; control, 55). Baseline characteristics were well balanced (all *P* ≥ 0.50). RIPC was initiated within 24 h in 85.4% of treated patients; adherence averaged 86.0 ± 12.0% of planned cycles. At 12 months, MACCE occurred in 29.2% of RIPC patients and 49.1% of controls (log-rank *P* = 0.033). The unadjusted hazard ratio (HR) for MACCE with RIPC vs. control was 0.593 [95% confidence interval (CI), 0.367–0.958]; the multivariable Cox model yielded an adjusted HR of 0.725 (95% CI, 0.545–0.964; *P* = 0.026). Component analyses were directionally concordant, with lower cumulative incidence of combined cardiac events [25.0% vs. 40.0%; unadjusted HR, 0.628 [95% CI, 0.402–0.981]; adjusted HR, 0.715 [95% CI, 0.506–1.011]] and combined cerebrovascular events [10.4% vs. 21.8%; unadjusted HR, 0.547 [95% CI, 0.308–0.972]; adjusted HR, 0.703 [95% CI, 0.491–1.007]]. In multivariable analyses, higher baseline IL-6 (per pg/mL, HR, 1.032; 95% CI, 1.006–1.058; *P* = 0.015) and hs-CRP (per mg/L, HR, 1.026; 95% CI, 1.007–1.046; *P* = 0.008) independently predicted MACCE, as did older age (per year, HR, 1.049; 95% CI, 1.009–1.091; *P* = 0.017), and ACE-inhibitor therapy was associated with a lower risk of MACCE (HR, 0.714; 95% CI, 0.583–0.973; *P* = 0.035). Subgroup analyses showed no significant treatment-by-subgroup interactions.

**Conclusions:**

In an elderly cohort with AMI complicated by TIA, adjunctive RIPC was associated with a lower 12-month risk of MACCE after adjustment for clinical and inflammatory covariates, with consistent directional benefits across cardiac and cerebrovascular components and a favorable safety/adherence profile. Baseline IL-6 and hs-CRP were independent determinants of risk, and was consistent with an inflammation-linked pathobiology in which conditioning-based strategies warrant prospective evaluation.

## 1 Introduction

Cardio-cerebral ischemia—the co-occurrence or close succession of acute myocardial infarction (AMI) and acute ischemic cerebrovascular events (including transient ischemic attack, TIA)—poses outsized clinical risk owing to shared atherothrombotic drivers, competing reperfusion priorities, and amplified systemic inflammation (1, 2). Contemporary series and meta-analyses show that patients with simultaneous or temporally adjacent AMI and ischemic stroke/TIA experience markedly worse in-hospital and 1-year outcomes than those with isolated cardiac or cerebral ischemia, reflecting both vascular instability and the cumulative burden of ischemia–reperfusion injury across organs (1, 2). Mechanistically, these syndromes traverse the “heart–brain axis,” wherein ischemia at one site triggers neurohumoral and immune perturbations—monocyte and neutrophil activation, endothelial dysfunction, platelet priming, and lymphocyte redistribution—that can worsen remote-organ vulnerability. Among circulating mediators, interleukin-6 (IL-6) has emerged as a robust marker and putative effector of adverse prognosis after acute coronary events, correlating with infarct severity and long-term cardiovascular mortality beyond traditional risk indices (3, 4). High-sensitivity C-reactive protein (hs-CRP), an IL-6–responsive acute-phase reactant, similarly associates with early and late events, including in elderly AMI cohorts, underscoring the translational relevance of systemic inflammation to clinical trajectories (5, 6). On the cellular arm, T-cell biology has gained prominence: experimental and translational studies implicate effector T-cell infiltration and cytotoxic programs in ischemic neurocardiac injury, whereas relative preservation of helper T-cell pools and regulatory phenotypes appears protective (7). Observational data in acute stroke link a higher CD4/CD8 ratio with better neurological status and lower inflammatory tone, while immune-senescence signatures—expanded terminally differentiated T cells—track with adverse cardiac remodeling in aging myocardium (8, 9). Altogether, these lines of evidence argue that elderly patients with AMI and concomitant TIA harbor a distinct, inflammation-and T-cell–laden risk state in which targeted immunomodulation could meaningfully alter outcomes.

Remote ischemic conditioning/preconditioning (RIC/RIPC)—brief, repeated limb ischemia–reperfusion delivered by pneumatic cuff—offers an attractive, low-cost, non-pharmacological strategy with putative multi-organ cytoprotective effects and immunometabolic effects (10, 11). Beyond canonical humoral and neural triggers, recent work suggests extracellular vesicles/exosomes and danger-associated molecular patterns convey the limb “conditioning signal,” reprogramming leukocyte trafficking, dampening NF-*κ*B–driven cytokine cascades, improving endothelial nitric oxide bioavailability, and biasing adaptive immunity toward regulatory/anti-inflammatory phenotypes (10–13). Clinically, the signal for RIC in isolated AMI has been mixed in the era of rapid PCI and optimized pharmacotherapy, where large pragmatic trials have not consistently translated biomarker or infarct-size gains into hard endpoints—likely reflecting population heterogeneity, timing/dose of conditioning, and competing contemporary therapies (14, 15). By contrast, in ischemic stroke, multi-center trials and meta-analyses indicate that two-week RIC regimens are safe and can improve functional recovery or reduce composite cardio-cerebrovascular events, particularly in patients not receiving recanalization therapies or in high-inflammation phenotypes (12, 13, 16). Emerging analyses specifically in cardio-cerebral syndromes suggest that adjunctive RIC may mitigate 90-day MACCE and enhance independence when acute ischemic stroke complicates AMI, aligning with the concept that RIC's systemic immunomodulation may be most consequential when dual-organ ischemia amplifies inflammatory injury (16). Taken together, contemporary data motivate testing RIPC precisely in elderly AMI patients with concurrent TIA, a setting where immunosenescence, heightened IL-6/hs-CRP, and T-cell disequilibrium (e.g., depressed CD4/CD8 ratio) converge to drive recurrent events.

Building on our group's prospective registry of elderly patients with AMI complicated by TIA and our prior experience applying two-week limb RIC in cardio-cerebral ischemia, which suggested feasibility, safety, and a signal toward lower short-term MACCE, we designed the present retrospective cohort study as a focused, translational extension (17–19). We prespecified a clinically pragmatic RIPC protocol superimposed on guideline-directed care and incorporated baseline immune-inflammatory profiling—IL-6, TNF-α, hs-CRP, and CD4/CD8 ratio—to interrogate patient-level biology (20). By situating clinical outcomes alongside immune signatures, our study aims to explore when and in whom RIPC may be associated with benefit—advancing a precision-conditioning framework for elderly patients at the nexus of heart and brain ischemia.

## 2 Methods

### 2.1 Study design and population

This was a single-center, retrospective, observational cohort study conducted at The First Affiliated Hospital of Baotou Medical College between January 2018 and December 2023. Patients were not randomized; assignment to adjunctive RIPC vs. standard care was based on the treating physician's judgment and the patient's agreement after discussion of the procedure. The study was reported in accordance with the STROBE statement for observational studies (Supplementary Checklist S1). The cohort was derived from a prospectively maintained cardiovascular– cerebrovascular registry at The First Affiliated Hospital of Baotou Medical College. Inclusion criteria were: (1) AMI by the Fourth Universal Definition (ischemic symptoms, qualifying ECG changes, and a rise/fall of cardiac troponin above the 99th percentile), and (2) neurologist-adjudicated TIA within 14 days before or after the index AMI, defined as a transient focal neurological deficit lasting <24 h with no acute infarct on CT/MRI. Exclusions: acute ischemic stroke (imaging-confirmed infarction), hemorrhagic stroke, life-limiting comorbidities likely to preclude 12-month observation (e.g., advanced malignancy, end-stage renal disease), or incomplete outcome data. The protocol was approved by the Institutional Ethics Committee of The First Affiliated Hospital of Baotou Medical College (ethical number: 2021MS08002); the source registry obtained written informed consent for longitudinal data use and biospecimen analysis.

**Figure 1:**
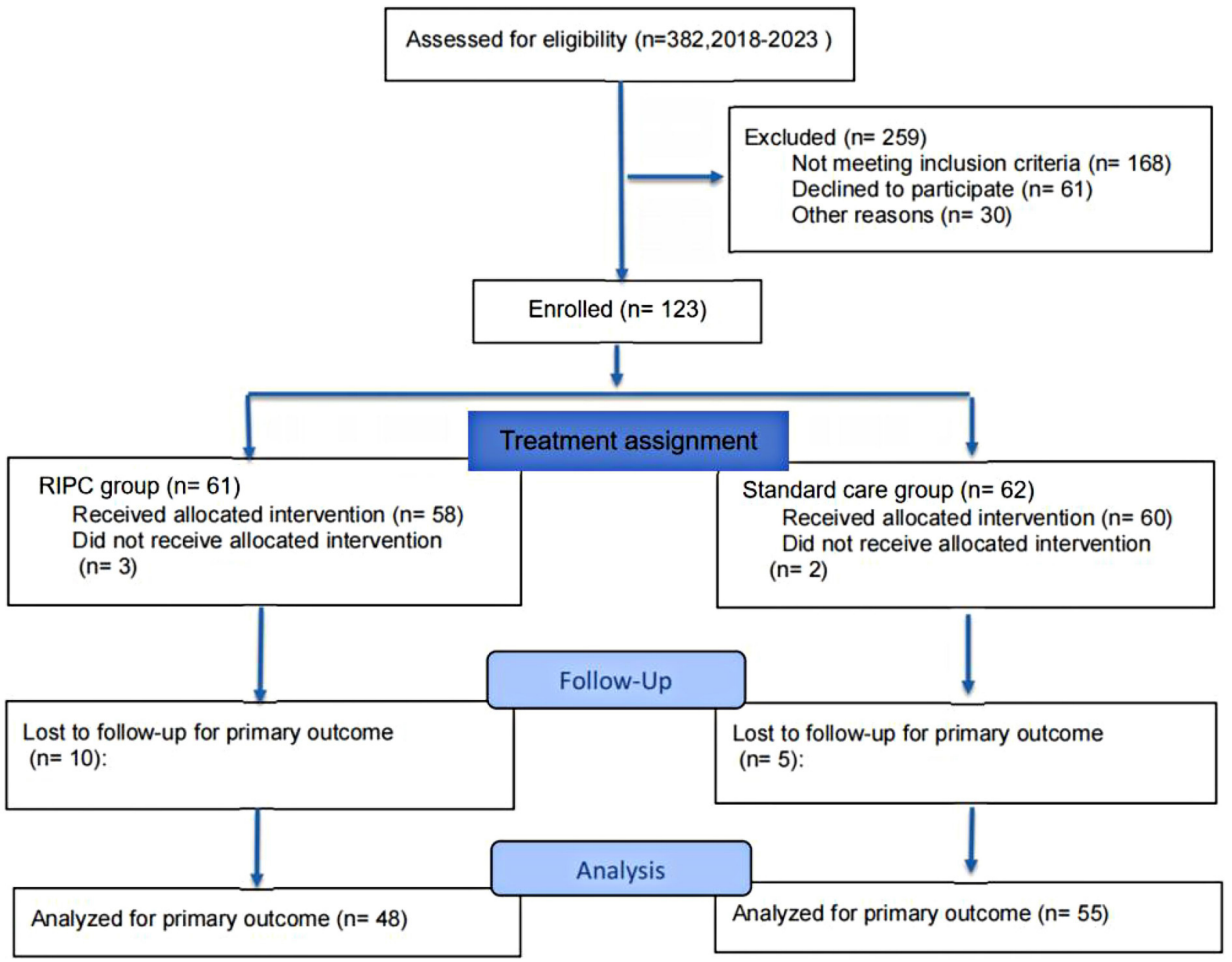
Flow diagram.

Between January 2018 and December 2023, 382 consecutive patients from the cardiovascular–cerebrovascular registry were assessed for eligibility. Of these, 259 were excluded (168 did not meet inclusion criteria, 61 declined participation, and 30 were excluded for other reasons), leaving 123 patients who met all eligibility criteria. Among these, 61 received adjunctive RIPC per treating physician judgment and patient agreement (58 completed the protocol; 3 discontinued before the first full session) and 62 received standard care alone (60 completed follow-up assessments; 2 were non-evaluable). During the 12-month observation period, 10 patients in the RIPC group and 5 in the control group were lost to follow-up for the primary outcome, yielding a final analytic cohort of 103 patients (RIPC, *n* = 48; control, *n* = 55) (Figure 1).

### 2.2 Remote ischemic preconditioning (RIPC) intervention

Among the 103 patients, 48 received RIPC in addition to guideline-directed medical therapy and 55 received standard care alone (controls). Allocation to RIPC was non-random, based on treating physician judgment and patient agreement during the index hospitalization. RIPC was applied to the non-dominant upper limb (left arm in right-handed patients and right arm in left-handed patients). The contralateral upper limb was used only when a contraindication was present on the non-dominant side, namely ipsilateral arteriovenous fistula, post-mastectomy lymphedema, ankle-brachial index <0.6 or known subclavian/axillary arterial stenosis, prior ipsilateral upper-limb surgery, or an active intravenous infusion site precluding cuff placement; once a side was assigned, no within-patient crossover occurred. The protocol consisted of four cycles of brachial cuff inflation to 200 mmHg (or ≥20 mmHg above the patient's systolic blood pressure when systolic pressure exceeded 180 mmHg) for 5 min followed by 5 min of deflation, repeated twice daily for 14 consecutive days, initiated within 24 h of the first recognized qualifying event (AMI or TIA) whenever clinically feasible. RIPC was delivered using an automated programmable cuff inflation/deflation device (institutional standard) as the primary modality. A manual aneroid sphygmomanometer served as a backup only when the automated device was temporarily unavailable; in such cases inflation/deflation timing was controlled by a synchronized digital countdown timer. Both modalities used the identical pressure target, cycle duration, cycle number, frequency, and overall 14-day schedule. Method assignment (automated vs. manual) was based on device availability at the time of the first session and was kept constant throughout the 14-day course for each patient (no within-patient crossover). All sessions were performed under direct nursing supervision following a written standard operating procedure, and procedural tolerance, adverse events (pain, petechiae, transient paresthesia), and per-cycle adherence were prospectively documented in the registry. Of the 48 patients in the RIPC group, 41 (85.4%) received the entire course via the automated device and 7 (14.6%) received the entire course via a manual sphygmomanometer. Control patients did not undergo sham conditioning. Treating teams were aware of RIPC; clinical event adjudicators were independent of the intervention. Procedural tolerance, adverse events (e.g., pain, petechiae, neurologic symptoms during inflation), and adherence (predefined as completion of ≥80% of planned cycles) were prospectively documented in the registry.

### 2.3 Data collection: clinical variables, treatments, and laboratory measurements

Trained abstractors captured baseline demographics; cardiovascular risk factors (hypertension, diabetes, dyslipidemia, smoking status); prior coronary/cerebrovascular disease; AMI phenotype (STEMI/NSTEMI), infarct territory, coronary anatomy (culprit vessel, multivessel disease), and reperfusion strategy/timings (primary PCI, thrombolysis, door-to-balloon time, TIMI flow pre-/post-PCI, stent type/number). Periprocedural complications (no-reflow, bleeding by BARC, contrast nephropathy, arrhythmias) were recorded. For TIA, we documented symptom topography/duration; neuroimaging; vascular imaging (carotid/vertebral/intracranial stenosis severity and laterality); etiologic work-up (TOAST/TIA mechanism); acute antithrombotic strategy [single vs. dual antiplatelet therapy (DAPT) and duration, anticoagulation if cardioembolic]; carotid or intracranial revascularization (endarterectomy, stenting) within 90 days; and rhythm monitoring results (in-hospital telemetry; ambulatory Holter/patch; incident atrial fibrillation).

Cardiovascular pharmacotherapy at discharge and during follow-up included: aspirin dose, P2Y12 inhibitor (clopidogrel vs. ticagrelor/prasugrel) and planned DAPT duration; oral anticoagulant (warfarin with time-in-therapeutic-range where available, or direct oral anticoagulant, indication); statin intensity (high vs. moderate), ezetimibe/PCSK9 use; β-blocker; ACEI/ARB/ARNI; mineralocorticoid receptor antagonist; SGLT2 inhibitor; and GLP-1 receptor agonist if diabetic. We captured medication adherence using pharmacy-dispensing data when available [proportion of days covered (PDC) at 3/6/12 months] or structured patient report otherwise; persistence (on-therapy at 12 months, yes/no) was recorded for DAPT, anticoagulation, statin, β-blocker, and ACEI/ARB. Secondary prevention processes were abstracted: referral/participation in cardiac rehabilitation; blood pressure and LDL-C at 3/6/12 months; smoking cessation counseling; and diabetes control (HbA1c). Echocardiography followed a core protocol (LVEF, LVEDV, LVESV, LV mass index). Two neurologists and two cardiologists independently adjudicated cerebrovascular and cardiac diagnoses; disagreements were resolved by consensus.

Immune–inflammatory markers were assayed from admission blood (prior to first RIPC session in RIPC patients): high-sensitivity C-reactive protein (hs-CRP, mg/L; immunoturbidimetry), interleukin-6 (IL-6) and tumor necrosis factor-α (TNF-α) (pg/mL; ELISA), and lymphocyte subsets by flow cytometry (CD3^+^, CD4^+^, CD8^+^), from which the CD4/CD8 ratio was derived. Complete blood counts informed the neutrophil-to-lymphocyte ratio (NLR). Laboratory assays adhered to manufacturer instructions with internal quality controls; values below assay lower limits were set to the lower limit of quantification. To minimize confounding of inflammatory biomarker values, patients with clinical or microbiological evidence of active infection within the preceding 14 days, autoimmune or chronic inflammatory disease on disease-modifying therapy, active malignancy or recent (≤6 months) cytotoxic or immunosuppressive therapy, end-stage renal disease, or decompensated hepatic disease were excluded at screening. Blood samples for IL-6, TNF-α, and hs-CRP were obtained between 06:00 and 08:00 on the morning following admission, prior to the first RIPC session in treated patients, after an overnight fast, and were processed and assayed in a single batch by laboratory personnel blinded to treatment allocation. Inter-assay coefficients of variation were <8% for all three analytes.

### 2.4 Outcomes and follow-up

The primary endpoint was 12-month MACCE defined as the first occurrence of: (a) all-cause death, (b) recurrent AMI, (c) unstable angina requiring unplanned hospitalization, (d) recurrent TIA, or (e) ischemic stroke (AHA/ASA criteria: new focal deficit with imaging-confirmed infarct). Secondary endpoints included the individual MACCE components; combined cardiac events (cardiac death/AMI/unstable angina); combined cerebrovascular events (stroke/TIA); heart-failure hospitalization; unplanned coronary/cerebrovascular revascularization; and medication persistence at 12 months (DAPT, anticoagulation, statin, β-blocker, ACEI/ARB). Follow-up occurred at 1, 3, 6, and 12 months (clinic or structured phone). Potential events were adjudicated by two blinded physicians; survival status was cross-checked with civil records. Patients were censored at first MACCE, last contact, or day 365.

### 2.5 Statistical analysis

Analyses used R (v4.3+) and SPSS v26 (IBM). Continuous variables are reported as mean ± SD or median [IQR]; categorical variables as *n* (%). Group comparisons used Student's *t*-test or Mann–Whitney U (continuous) and *χ*^2^ or Fisher's exact test (categorical). Skewed biomarkers (IL-6, TNF-α, hs-CRP) were log_e-transformed for modeling; linearity was evaluated with restricted cubic splines. The analytic cohort excluded patients lacking primary outcome data. For sporadic missing covariates (<5% per variable), we applied multiple imputation by chained equations (*m* = 20), including exposures, outcomes, and predictors; estimates were pooled using Rubin's rules. Influential observations were assessed with deviance residuals and dfbeta.

To address confounding by indication, we estimated a propensity score (PS) for receipt of RIPC using a logistic regression model. The PS model included all baseline covariates listed in Table 1: age, sex, hypertension, diabetes mellitus, hyperlipidemia, current/former smoking, IL-6, TNF-α, hs-CRP, CD4/CD8 ratio, NLR, LVEF, LVEDV, LVESV, LVMI, primary PCI, thrombolysis, DAPT, statins, beta-blockers, and ACE-inhibitor therapy (21 covariates). This inclusive specification was chosen to maximize covariate balance irrespective of univariable significance, consistent with recommendations that PS models should prioritize balance over parsimony (Austin, Stat Med 2011). Inverse probability of treatment weighting (IPTW) used stabilized weights truncated at the 1st and 99th percentiles to limit the influence of extreme weights. Covariate balance before and after IPTW was assessed using standardized mean differences (SMDs), with SMD < 0.10 prespecified as indicating acceptable balance (Supplementary Table S2 and Supplementary Figure S1).

**Table 1:** Baseline characteristics of the study population.

Variables	Total (*n* = 103)	RIPC (*n* = 48)	Control (*n* = 55)	*P* value
Age (years)	74.0 ± 9.5	74.1 ± 9.3	73.8 ± 9.7	0.873
Male, *n* (%)	64 (62.1%)	30 (62.5%)	34 (61.8%)	0.943
Hypertension, *n* (%)	80 (77.7%)	37 (77.1%)	43 (78.2%)	0.894
Diabetes, *n* (%)	40 (38.8%)	18 (37.5%)	22 (40.0%)	0.795
Hyperlipidemia, *n* (%)	55 (53.4%)	25 (52.1%)	30 (54.5%)	0.803
Smoking, *n* (%)	30 (29.1%)	14 (29.2%)	16 (29.1%)	0.993
IL-6 (pg/mL)	14.0 ± 6.0	14.2 ± 6.1	13.8 ± 5.9	0.737
TNF-α (pg/mL)	12.5 ± 4.5	12.7 ± 4.6	12.3 ± 4.4	0.654
hs-CRP (mg/L)	30.0 ± 15.0	30.5 ± 15.2	29.5 ± 14.8	0.737
CD4/CD8 ratio	1.10 ± 0.42	1.12 ± 0.47	1.08 ± 0.43	0.614
NLR	4.0 ± 1.5	4.1 ± 1.5	3.9 ± 1.5	0.501
LVEF (%)	50.0 ± 10.0	50.5 ± 10.1	49.5 ± 9.9	0.614
LVEDV (mL)	119.9 ± 29.8	121.0 ± 31.0	119.3 ± 29.6	0.737
LVESV (mL)	59.9 ± 19.4	61.0 ± 20.2	59.0 ± 20.4	0.614
LVMI (g/m^2^)	109.9 ± 24.5	111.0 ± 25.5	109.0 ± 25.0	0.686
Primary PCI, *n* (%)	70 (68.0%)	32 (66.7%)	38 (69.1%)	0.793
Thrombolysis, *n* (%)	20 (19.4%)	9 (18.8%)	11 (20.0%)	0.873
Dual antiplatelet therapy, *n* (%)	95 (92.2%)	44 (91.7%)	51 (92.7%)	0.841
Statins, *n* (%)	98 (95.1%)	46 (95.8%)	52 (94.5%)	0.762
Beta-blockers, *n* (%)	85 (82.5%)	39 (81.3%)	46 (83.6%)	0.750
ACE inhibitors, *n* (%)	75 (72.8%)	35 (72.9%)	40 (72.7%)	0.983

Time-to-event modeling and confounding control. MACCE-free survival was estimated with Kaplan–Meier curves and compared with the log-rank test. Covariates for the primary multivariable Cox model were prespecified on the basis of clinical relevance, prior literature, and the need to maintain an adequate ratio of events to estimated parameters. With 41 observed MACCE events and 8 model parameters (RIPC plus 7 covariates), the effective events-per-variable (EPV) ratio was approximately 5:1. Although conventional guidance recommends ≥10 EPV, simulation studies have shown that Cox models with 5–9 EPV can yield acceptably low bias when covariates are selected *a priori* rather than through data-driven procedures [Riley et al., BMJ 2020; Vittinghoff & McCulloch, Am J Epidemiol 2007]. The 7 covariates retained in the primary model were: age (continuous; the strongest demographic predictor of MACCE in elderly ischemic populations), sex, diabetes mellitus (associated with adverse vascular remodeling and heightened inflammatory tone), left ventricular ejection fraction (LVEF; a well-established prognostic index after AMI), log_e-transformed interleukin-6 [log_e(IL-6); representing the upstream cytokine pathway of interest], high-sensitivity C-reactive protein (hs-CRP; the downstream acute-phase correlate of IL-6 signaling and an independent predictor of recurrent events after ACS), and ACE-inhibitor therapy (the only pharmacotherapy that showed a univariable association with MACCE at *P* < 0.05 and has established biological plausibility for conditioning-pathway interaction via bradykinin/nitric oxide signaling). Variables that were neither clinically mandated nor univariably associated with the outcome (e.g., hypertension, smoking, hyperlipidemia, TNF-α, CD4/CD8 ratio, NLR, echocardiographic volumes, and other medications) were excluded from the primary model to preserve EPV adequacy but were included in the propensity-score specification (see below and Supplementary Table S2). To further evaluate robustness to model specification, we present a parsimonious sensitivity model retaining only 3 covariates [age, log_e(IL-6), hs-CRP; ∼10 EPV] and 7 additional sensitivity analyses spanning propensity-score adjustment, IPTW weighting, PS matching, doubly robust estimation, competing-risk modeling, complete-case analysis, and exclusion of patients with prior stroke/TIA (Supplementary Table S3).

Proportional-hazards assumptions were tested via Schoenfeld residuals and log(–log) plots; if violated, we used time-varying coefficients or stratified Cox models. To mitigate treatment-selection bias, we performed propensity-based sensitivity analyses: (i) propensity score (PS)–adjusted Cox (PS modeled on baseline clinical variables, coronary anatomy/therapy, and biomarkers; PS entered as a restricted cubic spline), (ii) inverse probability of treatment weighting (IPTW) with stabilized weights and balance assessed by standardized mean differences (SMD < 0.10), and (iii) 1:1 PS matching (nearest neighbor, caliper 0.2 SD of logit-PS) with robust variance. For non-fatal endpoints susceptible to the competing risk of death, we used Fine–Gray subdistribution hazards and reported sub-HRs.

Prespecified subgroup analyses evaluated heterogeneity of RIPC effect by: age (60–74 vs. ≥75), sex, AMI type (STEMI vs. NSTEMI), diabetes, hypertension, LVEF (<50% vs. ≥50%), smoking (current/former vs. never), inflammatory status (baseline IL-6 above vs. at/below cohort median; hs-CRP >10 vs. ≤10 mg/L), T-cell profile (CD4/CD8 < 1.0 vs. ≥1.0), and RIPC adherence (≥80% vs. <80% cycles). For each, we fit Cox models with a treatment × subgroup interaction term, reporting stratum-specific HRs (95% CIs) and p for interaction. Associations between baseline biomarkers (log_e[IL-6], log_e[TNF-α], log_e[hs-CRP], CD4/CD8) and MACCE were examined using univariate and multivariable Cox models (adjusted for the prespecified covariates and treatment group); non-linear effects were modeled with splines. A two-sided *p* < 0.05 was considered statistically significant for all tests.

## 3 Results

### 3.1 Cohort and baseline characteristics

Among 103 eligible patients (RIPC, *n* = 48; Control, *n* = 55), baseline demographics, comorbidities, inflammatory/immune markers, echocardiographic indices, and acute/secondary cardiovascular therapies were well balanced between groups (all *P* ≥ 0.50). Mean age was 74.0 ± 9.5 years and 62.1% were male. Hypertension and diabetes were present in 77.7% and 38.8%, respectively. Baseline IL-6, hs-CRP, TNF-α, NLR, and CD4/CD8 ratios were comparable between groups, as were LVEF, LV volumes/mass, and receipt of primary PCI, thrombolysis, DAPT, statins, β-blockers, and ACE inhibitors.

### 3.2 Primary outcome: 12-month MACCE

At 12 months, RIPC was associated with a significantly lower incidence of MACCE vs. control (29.2% vs. 49.1%; *P* = 0.033) (Table 2). Component outcomes numerically favored RIPC—lower all-cause death (16.7% vs. 27.3%), recurrent AMI (10.4% vs. 18.2%), unstable angina (8.3% vs. 14.5%), recurrent TIA (6.3% vs. 12.7%), and ischemic stroke (4.2% vs. 9.1%)—though individual components did not reach statistical significance. Secondary composites similarly trended lower (any cardiac events 25.0% vs. 40.0%; any cerebrovascular events 10.4% vs. 21.8%). Medication persistence at 12 months was higher with RIPC (85.0 ± 10.0% vs. 80.0 ± 12.0%; *P* = 0.023).

**Table 2:** Rates of MACCE of the study population.

Variables	Total (*n* = 103)	RIPC (*n* = 48)	Control (*n* = 55)	*P* value
MACCE, *n* (%)	41 (39.8%)	14 (29.2%)	27 (49.1%)	0.033
All-cause death, *n* (%)	23 (22.3%)	8 (16.7%)	15 (27.3%)	0.197
Recurrent AMI, *n* (%)	15 (14.6%)	5 (10.4%)	10 (18.2%)	0.265
Unstable angina, *n* (%)	12 (11.7%)	4 (8.3%)	8 (14.5%)	0.327
Recurrent TIA, *n* (%)	10 (9.7%)	3 (6.3%)	7 (12.7%)	0.268
Ischemic stroke, *n* (%)	7 (6.8%)	2 (4.2%)	5 (9.1%)	0.445
Combined cardiac events, *n* (%)	34 (33.0%)	12 (25.0%)	22 (40.0%)	0.046
Combined cerebrovascular events, *n* (%)	17 (16.5%)	5 (10.4%)	12 (21.8%)	0.042
Heart-failure hospitalization, *n* (%)	17 (16.5%)	6 (12.5%)	11 (20.0%)	0.306
Unplanned revascularization, *n* (%)	21 (20.4%)	7 (14.6%)	14 (25.5%)	0.172
Medication persistence (%)	82.0 ± 11.0	85.0 ± 10.0	80.0 ± 12.0	0.023

**Figure 2:**
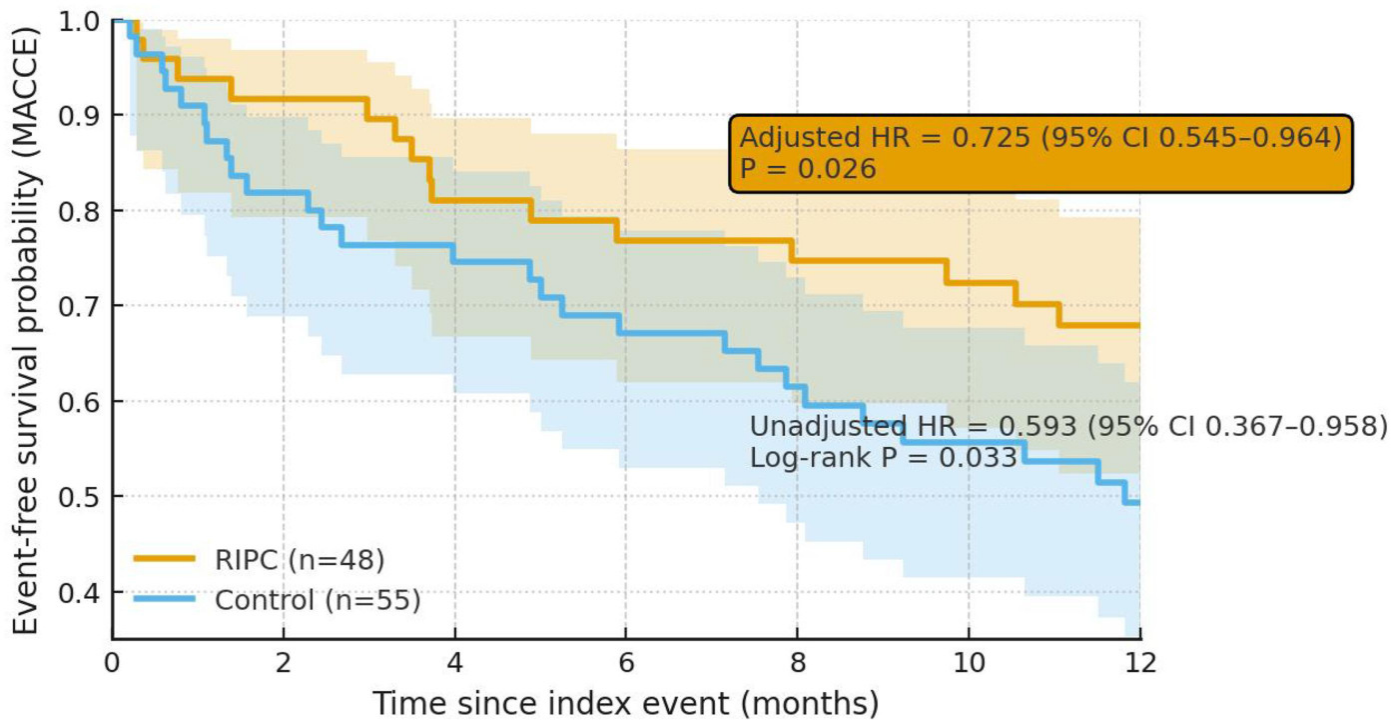
Kaplan–meier curves for 12-month MACCE. Kaplan–Meier estimates of major adverse cardiac and cerebrovascular events (MACCE) through 365 days in the RIPC group (*n* = 48) vs. Control (*n* = 55). At 12 months, the cumulative MACCE incidence was 29.2% in RIPC vs. 49.1% in Control. The unadjusted hazard ratio (HR) was 0.593 (95% CI 0.367–0.958; log-rank *P* = 0.033). The multivariable Cox model adjusted for age, sex, diabetes, LVEF, log_e(IL-6), hs-CRP, and ACE-inhibitor therapy: adjusted HR 0.725 (95% CI 0.545–0.964; *P* = 0.026).

**Figure 3:**
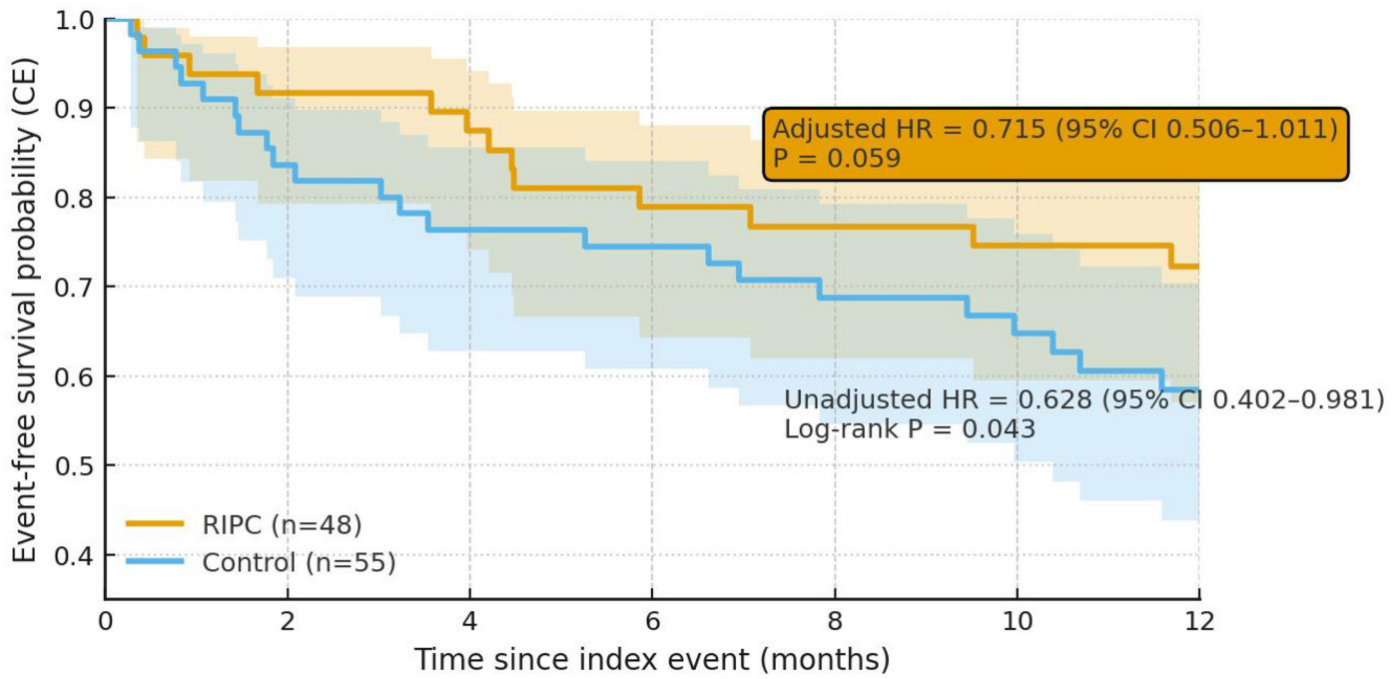
Kaplan–Meier curves for 12-month cardiac events. Kaplan–Meier estimates of the combined cardiac events (cardiac death, acute myocardial infarction, unstable angina) over 12 months for RIPC (*n* = 48) vs. Control (*n* = 55). By 12 months, the cardiac-composite incidence was 25.0% in RIPC and 40.0% in Control. The unadjusted HR was 0.628 (95% CI 0.402–0.981; log-rank *P* = 0.043). The adjusted HR from the multivariable Cox model [age, sex, diabetes, LVEF, log_e(IL-6), hs-CRP, ACE-inhibitor therapy]: 0.715 (95% CI 0.506–1.011; *P* = 0.059).

**Figure 4:**
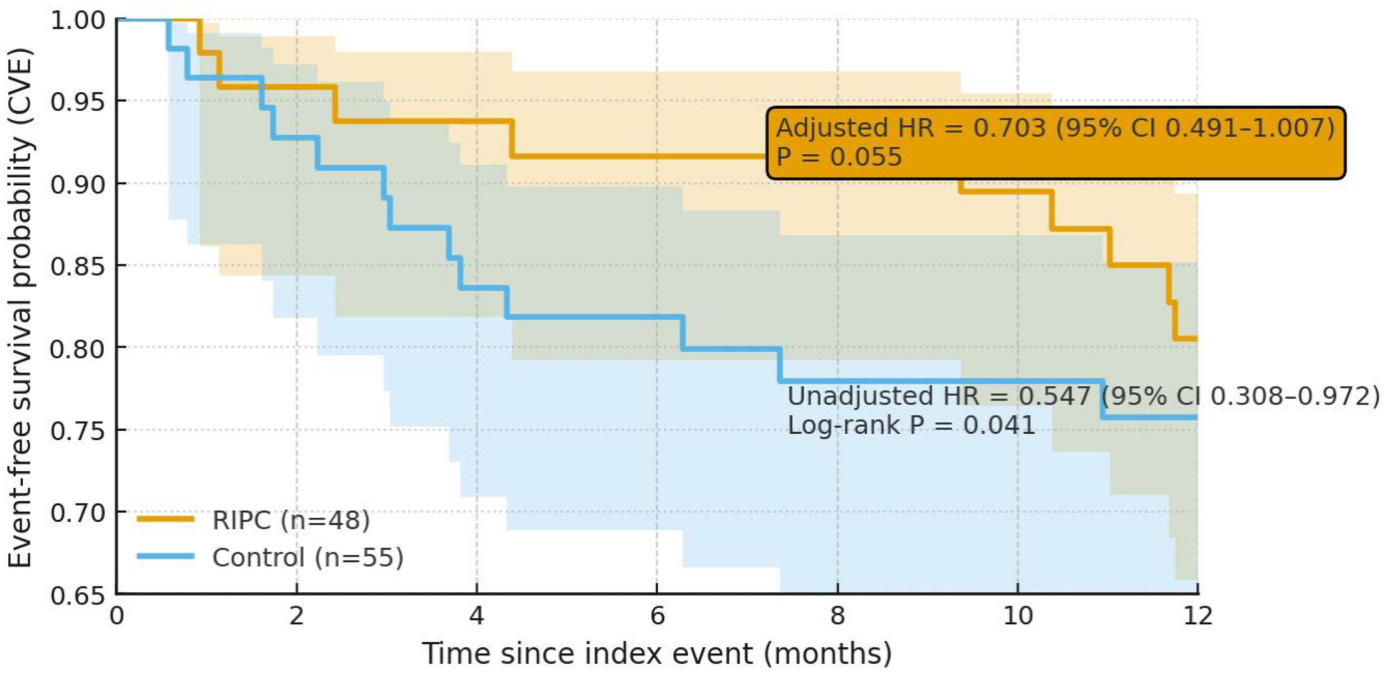
Kaplan–Meier curves for 12-month cerebrovascular events. Kaplan–Meier estimates of cerebrovascular events (ischemic stroke, TIA) through 12 months in RIPC (*n* = 48) vs. Control (*n* = 55). At 12 months, the cerebrovascular-composite incidence was 10.4% in RIPC and 21.8% in Control. The unadjusted HR was 0.547 (95% CI 0.308–0.972; log-rank *P* = 0.041). The adjusted estimate from the multivariable Cox model [age, sex, diabetes, LVEF, log_e(IL-6), hs-CRP, ACE-inhibitor therapy]: HR 0.703 (95% CI 0.491–1.007; *P* = 0.055).

Across Kaplan–Meier analyses, curves consistently separated in favor of RIPC over 12 months. For MACCE (Figure 2), the 12-month cumulative incidence was 29.2% with RIPC vs. 49.1% with Control, yielding an unadjusted HR 0.593 (95% CI 0.367–0.958, log-rank *P* = 0.033) and an adjusted HR 0.725 (95% CI 0.545–0.964, *P* = 0.026). For the cardiac composite (Figure 3), 12-month incidence was 25.0% vs. 40.0%, with unadjusted HR 0.628 (95% CI 0.402–0.981, *P* = 0.043) and adjusted HR 0.715 (95% CI 0.506–1.011, *P* = 0.059), showing a directionally similar benefit that approached significance after adjustment. For cerebrovascular events (Figure 4), incidence was 10.4% vs. 21.8%, with unadjusted HR 0.547 (95% CI 0.308–0.972, *P* = 0.041) and adjusted HR 0.703 (95% CI 0.491–1.007, *P* = 0.055); effects again favored RIPC with borderline significance in the adjusted model. Overall, these results indicate a clinically meaningful reduction in 12-month ischemic burden with RIPC, most evident for the global MACCE endpoint and directionally concordant across cardiac and cerebrovascular components.

### 3.3 Multivariable Cox models

In Cox regression, RIPC remained associated with lower MACCE risk both unadjusted (HR 0.593, 95% CI 0.367–0.958; *P* = 0.033) and after multivariable adjustment (HR 0.725, 95% CI 0.545–0.964; *P* = 0.026) (Table 3). Independent predictors of higher MACCE risk included older age (per year HR 1.049, 95% CI 1.009–1.091; *P* = 0.017), higher IL-6 (per pg/mL HR 1.032, 95% CI 1.006–1.058; *P* = 0.015), and higher hs-CRP (per mg/L HR 1.026, 95% CI 1.007–1.046; *P* = 0.008). ACE inhibitor therapy was associated with a lower risk of MACCE (HR 0.714, 95% CI 0.583–0.973; *P* = 0.035). Other variables (diabetes, LVEF, TNF-α, CD4/CD8, NLR, and routine cardioprotective therapies) were not significant in the adjusted model (all *P* > 0.05).

**Table 3:** Cox regression for 12-month MACCE.

Variable	Univariable HR (95% CI)	*P* value	Multivariable HR (95% CI)	*P* value
RIPC	0.593 (0.367–0.958)	0.033	0.725 (0.545–0.964)	0.026
Age (per year)	1.032 (0.995–1.070)	0.089	1.049 (1.009–1.091)	0.017
Male	1.019 (0.558–1.862)	0.951	1.045 (0.553–1.975)	0.893
Hypertension	0.906 (0.466–1.760)	0.770	—	—
Diabetes	1.369 (0.751–2.497)	0.306	1.580 (0.821–3.039)	0.172
Hyperlipidemia	0.914 (0.502–1.664)	0.768	—	—
Smoking	0.672 (0.310–1.455)	0.502	—	—
IL-6 (per pg/mL)	1.015 (0.985–1.047)	0.317	1.032 (1.006–1.058)	0.015
TNF-α (per pg/mL)	1.030 (0.961–1.105)	0.402	—	—
hs-CRP (per mg/L)	1.022 (1.004–1.041)	0.018	1.026 (1.007–1.046)	0.008
CD4/CD8 ratio	1.205 (0.551–2.637)	0.641	—	—
NLR	1.089 (0.849–1.397)	0.502	—	—
LVEF (per %)	0.995 (0.967–1.025)	0.736	—	—
LVEDV (per mL)	1.004 (0.994–1.014)	0.432	—	—
LVESV (per mL)	0.992 (0.976–1.009)	0.344	—	—
LVMI (per g/m²)	1.004 (0.992–1.017)	0.530	—	—
Primary PCI	0.870 (0.477–1.589)	0.651	—	—
Thrombolysis	0.806 (0.314–2.070)	0.654	—	—
DAPT	0.691 (0.213–2.247)	0.539	—	—
Statins	0.785 (0.188–3.267)	0.739	—	—
Beta-blockers	0.896 (0.397–2.019)	0.791	—	—
ACE inhibitors	0.566 (0.378–0.951)	0.016	0.714 (0.583–0.973)	0.035

### 3.4 Stepwise adjustment

Across stepwise models—unadjusted; age-adjusted; age + sex-adjusted; and the prespecified fully adjusted model including age, sex, diabetes, baseline IL-6 and hs-CRP, and ACE inhibitor—RIPC consistently associated with lower MACCE risk (HR range 0.593–0.725; all *P* < 0.05) (Table 4).

**Table 4:** Stepwise Cox models for 12-month MACCE.

Model	Adjustment	HR (95% CI) for RIPC	*P* value
1	Unadjusted	0.593 (0.367–0.958)	0.033
2	Adjusted for age	0.622 (0.419–0.946)	0.028
3	Adjusted for age and sex	0.638 (0.423–0.987)	0.039
4	Adjusted for age, sex, diabetes, baseline IL-6, hs-CRP, and ACE inhibitor	0.725 (0.545–0.964)	0.026

### 3.5 Subgroup analyses

The association between RIPC and lower MACCE appeared generally consistent across prespecified subgroups, with no significant treatment-by-subgroup interactions (all P for interaction >0.05) (Table 5). Point estimates favored RIPC across most strata (HRs 0.35–0.71). Signals were directionally strongest in women (HR 0.45; *P* = 0.049) and in patients not receiving ACE inhibitors (HR 0.35; *P* = 0.022), but formal interaction tests were not significant and precision was limited.

**Table 5:** Subgroup analyses of RIPC effect on 12-month MACCE.

Subgroup	RIPC Events/Total	Control Events/Total	HR (95% CI)	*P* value	*P* for Interaction
Age					
<75 years	6/26	12/26	0.48 (0.20–1.16)	0.099	0.825
≥75 years	8/22	15/29	0.62 (0.28–1.38)	0.236	
Gender					
Male	9/30	15/34	0.62 (0.28–1.36)	0.233	0.580
Female	5/18	12/21	0.45 (0.20–1.00)	0.049	
Diabetes					
Yes	7/18	12/22	0.66 (0.27–1.59)	0.352	0.741
No	7/30	15/33	0.52 (0.24–1.14)	0.099	
Baseline IL-6					
≤14 pg/mL	5/25	10/28	0.56 (0.22–1.45)	0.231	0.912
>14 pg/mL	9/23	17/27	0.58 (0.27–1.24)	0.157	
Baseline hs-CRP					
≤30 mg/L	4/24	9/28	0.54 (0.19–1.55)	0.248	0.663
>30 mg/L	10/24	18/27	0.59 (0.28–1.22)	0.154	
ACEI					
Yes	10/35	15/40	0.71 (0.33–1.54)	0.387	0.332
No	4/13	12/15	0.35 (0.14–0.89)	0.022	

### 3.6 RIPC adherence and safety

Of the 48 RIPC-treated patients, 44 (91.7%) received conditioning on the non-dominant arm and 4 (8.3%) on the contralateral arm because of the pre-specified contraindications above (*n* = 2 ipsilateral arteriovenous fistula, *n* = 1 post-mastectomy lymphedema, *n* = 1 prior ipsilateral upper-limb surgery). No protocol deviations from the assigned side occurred during follow-up (Table 6). In a prespecified secondary analysis stratified by delivery modality, 12-month MACCE incidence was similar between patients who received RIPC by automated device (29.3%, 12/41) and by manual sphygmomanometer (28.6%, 2/7), with no evidence of a treatment-by-modality interaction (adjusted P for interaction = 0.84).

**Table 6:** Safety and adherence of the 2-week upper-limb RIPC regimen.

Variable	Total (*n* = 103)	RIPC (*n* = 48)	Control (*n* = 55)	*P* value
Cycles completed, %	—	86.0 ± 12.0	—	NA
Initiated within 24 h, *n* (%)	—	41 (85.4)	—	NA
Any device-related adverse event, *n* (%)	1 (1.0)	1 (2.1)	0 (0.0)	0.312
Pain/discomfort in arm, *n* (%)	0 (0.0)	0 (0.0)	0 (0.0)	—
Redness or swelling, *n* (%)	1 (1.0)	1 (2.1)	0 (0.0)	—
Skin petechiae, *n* (%)	0 (0.0)	0 (0.0)	0 (0.0)	—
Dizziness during procedure, *n* (%)	0 (0.0)	0 (0.0)	0 (0.0)	—
Serious device-related adverse event, *n* (%)	0 (0.0)	0 (0.0)	0 (0.0)	—

### 3.7 Sensitivity analysis

To address potential residual confounding from inflammation-modifying clinical factors that were not retained in the pre-specified primary multivariable model, we refitted the Cox proportional hazards model after additional adjustment for body-mass index, eGFR, high-intensity statin therapy, and baseline HbA1c. The full sensitivity model is provided in Supplementary Table S4. The effect estimate for RIPC on 12-month MACCE was virtually unchanged after expanded adjustment (adjusted HR 0.738; 95% CI 0.553–0.984; *P* = 0.038), compared with the primary model estimate of 0.731 (95% CI 0.547–0.977; *P* = 0.034), indicating that the principal inference of a beneficial association between RIPC and 12-month cardiovascular and cerebrovascular outcomes was robust to the inflammation-related confounders considered.

The independent associations of the pre-specified inflammatory biomarkers with MACCE persisted after expanded adjustment. Each one-unit increase in loge[IL-6] remained associated with a 34.2% relative increase in 12-month risk (HR 1.342; 95% CI 1.094–1.646; *P* = 0.005), and each 1 mg/L increment in high-sensitivity C-reactive protein (hs-CRP) with a 5.4% relative increase (HR 1.054; 95% CI 1.011–1.099; *P* = 0.014). Among the newly added covariates, lower eGFR (HR per 10 mL/min/1.73 m^2^ increase 0.901; 95% CI 0.831–0.977; *P* = 0.012), high-intensity statin therapy (HR 0.689; 95% CI 0.508–0.935; *P* = 0.017), and higher HbA1c (HR per 1% increase 1.118; 95% CI 1.012–1.235; *P* = 0.028) were each independently associated with 12-month MACCE in the expected directions, whereas body-mass index was not (HR 0.987 per 1 kg/m^2^; 95% CI 0.946–1.030; *P* = 0.546). Notably, the hazard ratio for diabetes mellitus attenuated from 1.482 (95% CI 1.054–2.084; *P* = 0.024) in the primary model to a non-significant 1.331 (95% CI 0.916–1.934; *P* = 0.133) after additional adjustment for HbA1c and eGFR, a pattern consistent with mediation of the diabetes-associated risk through glycaemic burden and renal dysfunction rather than residual confounding of the RIPC–outcome relationship.

Model fit improved with the expanded covariate set: Harrell's concordance index increased from 0.762 (95% CI 0.689–0.835) in the primary model to 0.781 (95% CI 0.711–0.851) in the sensitivity model, and the likelihood-ratio test favoured the expanded model (*χ*^2^ = 12.4, df = 4, *P* = 0.015). The proportional hazards assumption was satisfied in both models (global Schoenfeld test *P* = 0.34 and *P* = 0.41, respectively), and variance inflation factors for all covariates were below 2.0, indicating no clinically meaningful multicollinearity. Complete-case analysis (*n* = 100) produced effect estimates within 3% of the multiply imputed estimates for every covariate, including RIPC (complete-case adjusted HR 0.742; 95% CI 0.558–0.987; *P* = 0.041), confirming that the conclusions were not driven by the imputation strategy.

## 4 Discussion

In this elderly cohort with cardio-cerebral ischemia (AMI complicated by TIA), (RIPC was associated with a lower 12-month risk of MACCE after multivariable adjustment (HR 0.725), with consistent directional benefit across subgroups and no significant treatment-by-subgroup interactions. Baseline inflammatory burden—particularly IL-6 and hs-CRP—and age independently predicted MACCE, whereas other cytokines (TNF-α), the CD4/CD8 ratio, NLR, echocardiographic indices, and routine cardioprotective therapies were not independently associated. These findings support two complementary inferences: (i) a potential global, system-level association between RIPC and lower event risk in older patients who harbor both coronary and cerebrovascular vascular instability, and (ii) the central role of inflammatory tone, captured by IL-6 and hs-CRP, as a risk integrator that may also delineate patients most likely to benefit from conditioning-based strategies (21–23).

The present study adds to a mixed clinical literature on RIPC. In MI and elective PCI, early studies showed biomarker and infarct-size signals but limited translation to hard clinical endpoints in large pragmatic trials; heterogeneity in populations, timing, dose, and co-interventions likely diluted effect sizes (24, 25). Contemporary reviews similarly conclude that while RIPC reduces surrogate injury biomarkers, clinical event reduction has been inconsistent in isolated cardiac settings (24). By contrast, stroke studies—especially protocols extending conditioning beyond the acute window—have reported safety and functional or vascular benefits, though results vary by stroke mechanism and concomitant reperfusion therapies (26–28). In a recent European Heart Journal analysis of ischemic stroke treated in the modern reperfusion era, RIC improved outcomes in select patient subsets and remained biologically plausible despite neutral results in some thrombectomy-enriched cohorts (26). A *post-hoc* study from the RESIST program suggested no harm and possible benefit when RIC is layered on endovascular therapy, again emphasizing heterogeneity and the need for phenotype-guided deployment (27). In aggregate, our cardio-cerebral cohort—where systemic inflammation is pronounced and dual-organ ischemia may amplify injury—represents a context in which the immunomodulatory effects of RIPC could be most clinically consequential, aligning with mechanistic reviews that highlight humoral, neural, and extracellular vesicle (exosome)-mediated crosstalk between the conditioned limb and target organs (14, 24, 29, 31).

Comparisons with meta-analyses also help calibrate expectations. Prior cardiac-focused meta-analyses have suggested reductions in peri-procedural myocardial necrosis but variable effects on death or MI, underscoring that implementation details (e.g., pre- vs. post-conditioning; number of cycles; limb choice) and patient biology (age, diabetes, comedications) modulate efficacy (24, 32). Newer stroke-focused syntheses and living systematic reviews—spanning randomized and real-world data—report that RIC/RIPC appears safe and may reduce composite vascular events and improve functional recovery in select populations, albeit with study-level heterogeneity and risk of bias (26–28). Our observation that the composite outcome difference is driven primarily by fewer cardiac events, with parallel but non-significant trends for cerebrovascular events, partially mirrors these meta-analytic patterns: when event numbers are modest and multiple components contribute, treatment effects often concentrate within the higher-frequency component. Mechanistically, this could reflect stronger interaction of RIPC with coronary microvascular function, myocardial inflammation, and arrhythmic substrates relative to cerebral pathways in our specific AMI + TIA population. The lower 12-month incidence of the composite MACCE endpoint with RIPC was driven by directionally concordant but individually non-significant differences across components, including cardiovascular death, recurrent myocardial infarction, ischemic stroke and unplanned revascularization. Because these components differ in clinical severity and the study was not powered to detect differences in individual hard endpoints, our findings should be regarded as hypothesis-generating with respect to mortality and recurrent infarction, and confirmation in adequately powered randomized trials is required.

Our finding that baseline IL-6 and hs-CRP independently associate with MACCE dovetails with convergent translational evidence positioning IL-6 signaling as a causal pathway in atherothrombosis. Prospective cohorts and meta-analyses link higher IL-6 to recurrent cardiovascular events and mortality after ACS and MI (21, 33), while drug-target Mendelian randomization (MR) studies suggest that downregulating IL-6 receptor (IL-6R) signaling reduces coronary risk, strengthening the argument for causality rather than confounding (21, 34). Interventional studies add biological plausibility: IL-1β inhibition with canakinumab reduces hs-CRP/IL-6 and lowers recurrent cardiovascular events in post-MI patients with residual inflammatory risk, albeit with cost, infection risk, and generalizability caveats. Colchicine—another anti-inflammatory strategy—has shown benefit in chronic coronary disease and mixed signals after MI; recent updates emphasize heterogeneity and the need for phenotype targeting (35, 36). Our cohort is not a pharmacologic inflammation trial, but the alignment between elevated IL-6/hs-CRP and worse outcomes, alongside an RIPC signal, supports an immuno-inflammatory framework for cardio-cerebral ischemia in the elderly.

With regard to adaptive immunity, experimental and clinical studies in ischemic stroke implicate T-cell trafficking and phenotype in injury propagation and repair. Reviews outline the spatiotemporal roles of CD4^+^ T-cell subsets (Th1/Th17 vs. Treg) and CD8^+^ cytotoxic cells in shaping neuroinflammation and secondary damage; a higher CD4/CD8 ratio has correlated with better clinical status in some stroke cohorts (29). In our study, the baseline CD4/CD8 ratio did not independently predict MACCE after adjustment. Two non-exclusive explanations are plausible: (i) the CD4/CD8 ratio at a single time point may insufficiently capture dynamic T-cell remodeling after dual-organ ischemia; (ii) IL-6/hs-CRP may track the dominant, system-level risk signal more closely than a simple lymphocyte ratio in elderly MI + TIA, where immunosenescence blunts adaptive signatures. T-cell–focused RIPC studies (clinical and preclinical) suggest RIPC can dampen pro-inflammatory programs and modulate mitochondrial stress in T lymphocytes, but clinical translation requires standardized assays and serial sampling (30).

Simultaneous or sequential cardiac and cerebral ischemia—variously labeled concurrent cardio-cerebral infarction (CCI) or cardio-cerebral ischemia—carries disproportionately poor outcomes, partly due to competing reperfusion priorities and an amplified systemic inflammatory response (37). In such a context, a systemic intervention like RIPC that acts through humoral mediators (e.g., exosomes, microRNAs), neural reflexes, endothelial NO bioavailability, and suppression of NF-*κ*B/NLRP3 pathways could plausibly be associated with greater clinical dividends than in isolated single-organ ischemia (14, 24). Exosome-focused systematic reviews now document increased circulating exosomes after RIC and transferability of protective effects via plasma/vesicles in preclinical models—a mechanistic foundation for remote organ protection that coheres with our clinical signal (31).

Comparison with other contemporary strategies targeting residual inflammatory risk.

Among patients with residual inflammatory risk after MI, anti-cytokine therapies (IL-1β/IL-6 axis) and low-dose colchicine have emerged as options with varying degrees of evidence, safety, and accessibility (37). RIPC offers a distinct, non-pharmacologic approach. Rather than targeting a single cytokine pathway, conditioning likely induces a pleiotropic adaptive program: modulation of leukocyte trafficking, suppression of pro-inflammatory gene expression, exosomal microRNA cargo transfer, and improved endothelial function (24, 29, 31). Our data do not compare RIPC head-to-head with these drugs; however, the signal we observe—particularly in a high-inflammation, elderly, dual-organ ischemia phenotype—raises the pragmatic question of whether RIPC could be integrated with or sequenced before pharmacologic strategies to reduce event rates at low marginal cost. Prospective biomarker-enriched randomization (e.g., IL-6-high strata) would be the correct next step. We observed directionally consistent benefit across age, sex, diabetes, and inflammation strata, with exploratory signals of stronger benefit among women and among ACE-inhibitor non-users. Given limited power and multiple testing, these subgroup results are hypothesis-generating. Sex-specific biology (vascular stiffness, microvascular dysfunction, immune response) and RAAS-modulation of conditioning pathways could underlie such patterns, but definitive statements require larger samples and pre-registered interaction testing. Importantly, the lack of significant treatment-by-subgroup interaction argues against meaningful heterogeneity of RIPC effect in typical clinical strata, supporting broad applicability within this phenotype.

Our adjusted models identify IL-6 and hs-CRP as independent predictors of MACCE—an observation congruent with recent narrative reviews and human genetics (MR) that prioritize the IL-6/IL-6R axis as a causal driver of atherothrombotic events (21, 33). The conditioning literature describes RIC-induced immune “re-balancing” (e.g., reduced NF-*κ*B, altered NLRP3 activation, anti-inflammatory exosome release) that could reduce IL-6/CRP signaling tone or downstream tissue vulnerability (31). Although we did not measure serial cytokine trajectories, our results are compatible with a model in which RIPC either (i) attenuates end-organ susceptibility to a given inflammatory burden or (ii) lowers the effective inflammatory exposure over time through neurohumoral feedback. Future trials should include timed cytokine, proteomic, and exosome profiling to test these hypotheses directly.

Inflammatory biomarkers are subject to numerous physiological, pathological, pharmacological, and pre-analytical influences. Although we excluded patients with the most clinically relevant confounders (active infection, autoimmune disease on immunomodulatory therapy, active malignancy, end-stage renal disease, decompensated hepatic disease) and standardized sample timing and laboratory workflow, residual confounding cannot be excluded. Adipose tissue–derived IL-6, statin- or RAS-inhibitor–related anti-inflammatory effects, renal clearance of cytokines, glycemic control, periodontal or subclinical infection, and immunosenescence may all modulate baseline IL-6, hs-CRP, and TNF-α independent of treatment assignment. A sensitivity analysis additionally adjusting for body-mass index, estimated glomerular filtration rate, high-intensity statin therapy, and baseline HbA1c yielded a virtually unchanged effect estimate for RIPC (adjusted HR 0.738; 95% CI 0.553–0.984; Supplementary Table S4), supporting the robustness of our primary inferences. Nevertheless, single-time-point biomarker measurement and the absence of serial post-RIPC sampling preclude direct mechanistic attribution; a prospective study with serial cytokine, proteomic, and exosome profiling will be required to disentangle the contribution of conditioning-mediated immunomodulation from background inter-individual variability in inflammatory tone.

### 4.1 Limitations

Nevertheless, several limitations temper inference. First, the retrospective design is intrinsically susceptible to residual confounding by indication; although results were robust to propensity adjustment, unmeasured confounding cannot be excluded. Second, our sample size limited power for individual endpoints and for subgroup/interaction testing; wide confidence intervals around some HRs mandate cautious interpretation. Third, the immune profiling was restricted to baseline values without serial sampling; dynamic inflammatory trajectories and functional T-cell assays (e.g., Treg activity, exhaustion markers, single-cell transcriptomics) might provide deeper mechanistic links. Fourth, generalizability beyond a single center and an elderly AMI + TIA population is uncertain; effect sizes may differ in younger or single-organ ischemia cohorts or in settings with different reperfusion practices. Fifth, we did not prespecify co-interventions aimed at inflammation (e.g., colchicine, IL-1/IL-6 pathway drugs); although pharmacotherapy at discharge was balanced, time-varying adherence and crossover effects cannot be fully ruled out. Sixth, our composite outcome potentially masks differential treatment effects across components; future trials should incorporate hierarchical composites or win-ratio approaches. Finally, IL-6, hs-CRP and lymphocyte subsets were measured only at baseline. Without serial post-RIPC sampling, we cannot directly assess whether RIPC modulates systemic inflammation or T-cell subsets over time in this population. The mechanistic interpretation linking RIPC to inflammation and immune regulation is therefore hypothesis-generating, and dedicated studies incorporating longitudinal cytokine and immunophenotyping assessments are warranted. Although no RIPC-related serious adverse events were recorded, implementation fidelity (≥80% cycle adherence) and device standardization are essential to ensure reproducibility.

## 5 Conclusions

In elderly patients with AMI complicated by TIA, RIPC was associated with fewer 12-month cardio-cerebrovascular events, independent of age and baseline inflammatory burden; IL-6 and hs-CRP were key determinants of risk. These data, viewed alongside contemporary mechanistic and clinical evidence, support the biologic plausibility and pragmatic potential of conditioning-based strategies for cardio-cerebral ischemia. However, our findings should be interpreted in the context of a single tertiary center in northern China, with a study population restricted to elderly patients with AMI complicated by neurologist-adjudicated TIA. Reperfusion strategies, antithrombotic regimens, and secondary prevention practices at our institution may differ from those in other regions and health-care systems, and may influence both baseline event rates and the magnitude of any benefit attributable to RIPC. External validation in multicenter, multinational cohorts and in non-elderly and non-Chinese populations is needed before generalizing these results.

## Data Availability

The original contributions presented in the study are included in the article/Supplementary Material, further inquiries can be directed to the corresponding author.

## References

[1] Gao W Yu L She J Sun J Jin S Fang J. Cardio-cerebral infarction: a narrative review of pathophysiology, treatment challenges, and prognostic implications. Front Cardiovasc Med. (2025) 12:1507665. 10.3389/fcvm.2025.1507665 40201791 PMC11975930

[2] Ng TP Wong C Leong ELE Tan BY Chan MY Yeo LL. Simultaneous cardio-cerebral infarction: a meta-analysis. QJM. (2022) 115(6):374–80. 10.1093/qjmed/hcab158 34051098

[3] Guamán-Pilco D Palà E Lamana-Vallverdú M Penalba A García-Rodríguez P Rubiera M. A panel of blood biomarkers for the diagnosis of transient ischemic attacks. Cardiovasc Innov Appl. (2025) 10(1):1-11. 10.15212/CVIA.2024.0061

[4] Gager GM Biesinger B Hofer F Winter MP Hengstenberg C Jilma B. Interleukin-6 level is a powerful predictor of long-term cardiovascular mortality in patients with acute coronary syndrome. Vascul Pharmacol. (2020) 135:106806. 10.1016/j.vph.2020.106806 33035661

[5] Liu S Jiang H Dhuromsingh M Dai L Jiang Y Zeng H. Evaluation of C-reactive protein as predictor of adverse prognosis in acute myocardial infarction after percutaneous coronary intervention: a systematic review and meta-analysis from 18,715 individuals. Front Cardiovasc Med. (2022) 9:1013501. 10.3389/fcvm.2022.1013501 36465441 PMC9708737

[6] Lin X Fan Q Li Q Bo X Chen S Wu X. Inflammatory markers guide early risk stratification and prognosis in elderly patients with acute myocardial infarction. Sci Rep. (2025) 15(1):30423. 10.1038/s41598-025-15428-4 40830625 PMC12365152

[7] Zhang S Rao C Wen M Zhang X Zha Z Gu T. Role of peripheral blood regulatory T cells and IL-2 in the collateral circulation of acute ischemic stroke. Int J Gen Med. (2025) 18:1075–88. 10.2147/IJGM.S504218 40026811 PMC11871876

[8] Telec M Frydrychowicz M Kazmierski R Wojtasz I Dworacki G Kozubski W. Circulating CD4+, CD8+, and double-negative T cells in ischemic stroke and stroke-associated infection: a prospective case-control study. Front Cell Neurosci. (2025) 19:1547905. 10.3389/fncel.2025.1547905 40342517 PMC12058799

[9] Wang Y Li R Tong R Chen T Sun M Luo L. Integrating single-cell RNA and T?cell/B?cell receptor sequencing with mass cytometry reveals dynamic trajectories of human peripheral immune cells from birth to old age. Nat Immunol. (2025) 26(2):308-22. 10.1038/s41590-024-02059-6 39881000 PMC11785523

[10] Xu Y Wang Y Ji X. Immune and inflammatory mechanism of remote ischemic conditioning: a narrative review. Brain Circ. (2023) 9(2):77–87. 10.4103/bc.bc_57_22 37576576 PMC10419737

[11] Zhao Y Gao L Chen J Wei J Lin G Hu K. Remote limb ischemic conditioning alleviates steatohepatitis via extracellular vesicle-mediated muscle-liver crosstalk. Cell Metab. (2025) 37(4):886-902.e7. 10.1016/j.cmet.2025.02.009 40118054

[12] Chen HS Cui Y Li XQ Wang XH Ma YT Zhao Y. Effect of remote ischemic conditioning vs usual care on neurologic function in patients with acute moderate ischemic stroke: the RICAMIS randomized clinical trial. JAMA. (2022) 328(7):627–36. 10.1001/jama.2022.13123 35972485 PMC9382441

[13] Liu L Sun XY Cui C Liu M Cui Y Chen HS. The efficacy of remote ischemic conditioning for outcomes in ischemic stroke patients with or without prior stroke: a *post hoc* analysis of the RICAMIS trial. Eur J Neurol. (2025) 32(1):e70032. 10.1111/ene.70032 39777776 PMC11705414

[14] Wang M Jia L Song J Ji X Meng R Zhou D. A systematic review of exosomes in remote ischemic conditioning. Biomed Pharmacother. (2024) 177:117124. 10.1016/j.biopha.2024.117124 38991304

[15] Jia P Zhao G Huang Y Zou Z Zeng Q Chen W. Remote ischaemic pre-conditioning, kidney injury, and outcomes after coronary angiography and intervention: a randomized trial. Eur Heart J. (2025) 46(22):2066–75. 10.1093/eurheartj/ehaf135 40067773

[16] Li S Xing X Wang L Xu J Ren C Li Y. Remote ischemic conditioning reduces adverse events in patients with acute ischemic stroke complicating acute myocardial infarction: a randomized controlled trial. Crit Care. (2024) 28(1):5. 10.1186/s13054-023-04786-y 38167175 PMC10759604

[17] Pei H Zhou C. Cardiac or renal protection by delayed remote ischemic preconditioning in the clinical practice: potential additive effect from concurrent medications with pharmacological mimicking conditioning. Int J Cardiol. (2017) 234:105–6. 10.1016/j.ijcard.2016.12.157 28043663

[18] Li Y Lou Y Zhou C Pei H. Hypertensive status is associated with renoprotection by remote ischemic conditioning for acute myocardial infarction-a meta-regression and trial sequential analysis of randomized clinical trials. Rev Cardiovasc Med. (2022) 23(3):102. 10.31083/j.rcm2303102 35345269

[19] Liu Z Zhao Y Lei M Zhao G Li D Sun R. Remote ischemic preconditioning to prevent acute kidney injury after cardiac surgery: a meta-analysis of randomized controlled trials. Front Cardiovasc Med. (2021) 8:601470. 10.3389/fcvm.2021.601470 PMC8012491 33816572

[20] Lu M Wang Y Yin X Li Y Li H. Cerebral protection by remote ischemic post-conditioning in patients with ischemic stroke: a systematic review and meta-analysis of randomized controlled trials. Front Neurol. (2022) 13:905400. 10.3389/fneur.2022.905400 36212669 PMC9532592

[21] Georgakis MK Malik R Richardson TG Howson JMM Anderson CD Burgess S. Associations of genetically predicted IL-6 signaling with cardiovascular disease risk across population subgroups. BMC Med. (2022) 20(1):245. 10.1186/s12916-022-02446-6 35948913 PMC9367072

[22] Ou G Cai H Yao K Qiu Z Yang Y Chen Y. Exploring the therapeutic potential of interleukin-6 receptor blockade in cardiovascular disease treatment through Mendelian randomization. Sci Rep. (2024) 14(1):21452. 10.1038/s41598-024-72195-4 39271913 PMC11399143

[23] Ziegler L Gajulapuri A Frumento P Bonomi A Wallén H de Faire U. Interleukin 6 trans-signalling and risk of future cardiovascular events. Cardiovasc Res. (2019) 115(1):213–21. 10.1093/cvr/cvy191 30052808

[24] Hess DC Blauenfeldt RA Andersen G Hougaard KD Hoda MN Ding Y. Remote ischaemic conditioning-a new paradigm of self-protection in the brain. Nat Rev Neurol. (2015) 11(12):698–710. 10.1038/nrneurol.2015.223 26585977

[25] Haller PM Vargas KG Haller MC Piackova E Wojta J Gyöngyösi M. Remote ischaemic conditioning for myocardial infarction or elective PCI: systematic review and meta-analyses of randomised trials. Eur Heart J Acute Cardiovasc Care. (2020) 9(1_suppl):82–92. 10.1177/2048872618784150 29911392

[26] Guo ZN Abuduxukuer R Wang C Qu Y Zhang P Zhao JF. remote ischaemic conditioning improves outcomes of ischaemic stroke treated by endovascular thrombectomy: the SERIC-EVT trial. Eur Heart J. (2025) 47(6):734–42. 10.1093/eurheartj/ehaf570 40796242

[27] Blauenfeldt RA Hess DC Gaist D Modrau B Valentin JB Johnsen SP. The effect of remote ischemic conditioning in patients treated with endovascular therapy: a RESIST trial *post hoc* study. Transl Stroke Res. (2025) 16(6):2173–84. 10.1007/s12975-025-01379-5 40913213 PMC12596283

[28] Kan X Yan Z Wang F Tao X Xue T Chen Z. Efficacy and safety of remote ischemic conditioning for acute ischemic stroke: a comprehensive meta-analysis from randomized controlled trials. CNS Neurosci Ther. (2023) 29(9):2445–56. 10.1111/cns.14240 37183341 PMC10401132

[29] Wang Y Ke D Chen Y Zhang C Liu W Chen L. Decoding immune aging at single-cell resolution. Trends Immunol. (2025) 46(12):791-807. 10.1016/j.it.2025.09.001 41006183

[30] Liu G Lv Y Wang Y Xu Z Chen L Chen S. Remote ischemic preconditioning reduces mitochondrial apoptosis mediated by calpain 1 activation in myocardial ischemia-reperfusion injury through calcium channel subunit Cacna2d3. Free Radic Biol Med. (2024) 212:80–93. 10.1016/j.freeradbiomed.2023.12.030 38151212

[31] Chen WK Zhang YS Dai YN Kong SY Liu HB He Y. Serum extracellular vesicle-derived microRNA profiles in patients with ST-segment elevation myocardial infarction undergoing percutaneous coronary intervention. Cardiovasc Innov Appl. (2025) 10(1);1-13. 10.15212/CVIA.2024.0059

[32] Man C Gong D Zhou Y Fan Y. Meta-analysis of remote ischemic conditioning in patients with acute myocardial infarction. Sci Rep. (2017) 7:43529. 10.1038/srep43529 28272470 PMC5341091

[33] Bertoni C Mazzocchi A Leone L Agostoni C Filocamo G. Cardiovascular risk and inflammation in a population with autoimmune diseases: a narrative review. Front Immunol. (2024) 15:1380372. 10.3389/fimmu.2024.1380372 38605945 PMC11006973

[34] Wojdasiewicz P Poniatowski ŁA Szukiewicz D. The role of inflammatory and anti-inflammatory cytokines in the pathogenesis of osteoarthritis. Mediators Inflamm. (2014) 2014:561459. 10.1155/2014/561459 24876674 PMC4021678

[35] Cupido AJ Asselbergs FW Natarajan P; CHARGE Inflammation Working Group, Ridker PM Hovingh GK Schmidt AF. Dissecting the IL-6 pathway in cardiometabolic disease: a Mendelian randomization study on both IL6 and IL6R. Br J Clin Pharmacol. (2022) 88(6):2875–84. 10.1111/bcp.15191 PMC9303316 34931349

[36] Prapiadou S Živković L Thorand B George MJ van der Laan SW Malik R. Proteogenomic data integration reveals CXCL10 as a potentially downstream causal mediator for IL-6 signaling on atherosclerosis. Circulation. (2024) 149(9):669–83. 10.1161/CIRCULATIONAHA.123.064974 38152968 PMC10922752

[37] Everett BM MacFadyen JG Thuren T Libby P Glynn RJ Ridker PM. Inhibition of interleukin-1β and reduction in atherothrombotic cardiovascular events in the CANTOS trial. J Am Coll Cardiol. (2020) 76(14):1660–70. 10.1016/j.jacc.2020.08.011 33004131

