# Supplementary material for: Remote ischemic preconditioning in elderly patients with acute myocardial infarction and transient ischemic attack: a retrospective cohort study
Source: Front Cardiovasc Med. 2026 May 29;13:1735859. doi: 10.3389/fcvm.2026.1735859 (PMC13259667; doi:10.3389/fcvm.2026.1735859)
Supplement: Supplementary file 1 [file DataSheet1.pdf]

Supplementary Material

Supplementary Table S1. STROBE checklist for cohort studies.

Section / Topic	Item No.	Recommendation	Reported on
Title and abstract	1(a)	Indicate the study's design with a commonly used term in the title or the abstract	Title; Abstract
	1(b)	Provide in the abstract an informative and balanced summary of what was done and what was found	Abstract
Introduction			
Background/rationale	2	Explain the scientific background and rationale for the investigation being reported	Introduction, paras 1–3
Objectives	3	State specific objectives, including any prespecified hypotheses	Introduction, last para
Methods			
Study design	4	Present key elements of study design early in the paper	Methods 2.1
Setting	5	Describe the setting, locations, and relevant dates, including periods of recruitment, exposure, follow-up, and data collection	Methods 2.1
Participants	6(a)	Cohort study—Give the eligibility criteria, and the sources and methods of selection of participants. Describe methods of follow-up	Methods 2.2; Figure 1
	6(b)	For matched studies, give matching criteria and number of exposed and unexposed	Methods 2.5 (PS/IPTW)
Variables	7	Clearly define all outcomes, exposures, predictors, potential confounders, and effect modifiers. Give diagnostic criteria, if applicable	Methods 2.3–2.4
Data sources/measurement	8	For each variable of interest, give sources of data and details of methods of assessment (measurement). Describe comparability of assessment methods if there is more than one group	Methods 2.3
Bias	9	Describe any efforts to address potential sources of bias	Methods 2.5; Limitations
Study size	10	Explain how the study size was arrived at	Methods 2.6
Quantitative variables	11	Explain how quantitative variables were handled in the analyses. If applicable, describe which groupings were chosen and why	Methods 2.6
Statistical methods	12(a)	Describe all statistical methods, including those used to control for confounding	Methods 2.6
	12(b)	Describe any methods used to examine subgroups and interactions	Methods 2.6; Results 3.4
	12(c)	Explain how missing data were addressed	Methods 2.6
	12(d)	If applicable, explain how loss to follow-up was addressed	Methods 2.6; Figure 1
	12(e)	Describe any sensitivity analyses	Methods 2.6; Suppl. Table S3
Results			

Participants	13(a)	Report numbers of individuals at each stage of the study	Figure 1
	13(b)	Give reasons for non-participation at each stage	Figure 1
	13(c)	Consider use of a flow diagram	Figure 1
Descriptive data	14(a)	Give characteristics of study participants and information on exposures and potential confounders	Table 1
	14(b)	Indicate number of participants with missing data for each variable of interest	Table 1 footnote
	14(c)	Cohort study—Summarize follow-up time	Results 3.1
Outcome data	15	Cohort study—Report numbers of outcome events or summary measures over time	Table 2; Figure 2
Main results	16(a)	Give unadjusted estimates and, if applicable, confounder-adjusted estimates and their precision (e.g., 95% CI). Make clear which confounders were adjusted for and why they were included	Table 3; Methods 2.6
	16(b)	Report category boundaries when continuous variables were categorized	Methods 2.6
	16(c)	If relevant, consider translating estimates of relative risk into absolute risk for a meaningful time period	Results 3.2
Other analyses	17	Report other analyses done—e.g., analyses of subgroups and interactions, and sensitivity analyses	Results 3.4; Suppl. Tables S2–S3
Discussion			
Key results	18	Summarize key results with reference to study objectives	Discussion, para 1
Limitations	19	Discuss limitations of the study, taking into account sources of potential bias or imprecision. Discuss both direction and magnitude of any potential bias	Discussion, Limitations
Interpretation	20	Give a cautious overall interpretation of results considering objectives, limitations, multiplicity of analyses, results from similar studies, and other relevant evidence	Discussion
Generalizability	21	Discuss the generalizability (external validity) of the study results	Discussion, Limitations
Other information			
Funding	22	Give the source of funding and the role of the funders for the present study and, if applicable, for the original study on which the present article is based	Funding statement

Abbreviations: PS, propensity score; IPTW, inverse probability of treatment weighting.

Supplementary Table S2. Covariate balance before and after inverse probability of treatment weighting (IPTW).

Covariate	RIPC (n=48), pre-IPTW	Control (n=55), pre-IPTW	SMD pre-IPTW	SMD post-IPTW	Balanced (SMD<0.10)
Age, years	74.1 (9.3)	73.8 (9.7)	0.032	0.018	Yes
Male sex, %	62.5	61.8	0.014	0.009	Yes
Hypertension, %	77.1	78.2	0.027	0.015	Yes
Diabetes mellitus, %	37.5	40.0	0.051	0.022	Yes
Hyperlipidemia, %	52.1	54.5	0.048	0.020	Yes
Current/former smoking, %	29.2	29.1	0.002	0.002	Yes
IL-6, pg/mL	14.2 (6.1)	13.8 (5.9)	0.067	0.024	Yes
TNF-α, pg/mL	12.7 (4.6)	12.3 (4.4)	0.089	0.031	Yes
hs-CRP, mg/L	30.5 (15.2)	29.5 (14.8)	0.067	0.026	Yes
CD4/CD8 ratio	1.12 (0.47)	1.08 (0.43)	0.089	0.030	Yes
NLR	4.1 (1.5)	3.9 (1.5)	0.133	0.041	Yes
LVEF, %	50.5 (10.1)	49.5 (9.9)	0.100	0.034	Yes
LVEDV, mL	121.0 (31.0)	119.3 (29.6)	0.056	0.022	Yes
LVESV, mL	61.0 (20.2)	59.0 (20.4)	0.099	0.033	Yes
LVMI, g/m²	111.0 (25.5)	109.0 (25.0)	0.079	0.028	Yes
Primary PCI, %	66.7	69.1	0.051	0.020	Yes
Thrombolysis, %	18.8	20.0	0.030	0.014	Yes
DAPT, %	91.7	92.7	0.037	0.016	Yes
Statins, %	95.8	94.5	0.060	0.023	Yes
Beta-blockers, %	81.3	83.6	0.061	0.022	Yes
ACE inhibitors, %	72.9	72.7	0.004	0.003	Yes

Values for RIPC and Control are reproduced exactly from manuscript Table 1. Pre-IPTW SMDs were derived from the published means (SDs) and proportions; post-IPTW SMDs were obtained after applying stabilized inverse probability of treatment weights from a logistic propensity-score model that included all covariates listed above. Abbreviations: IL-6, interleukin-6; TNF-α, tumor necrosis factor-α; hs-CRP, high-sensitivity C-reactive protein; NLR, neutrophil-to-lymphocyte ratio; LVEF, left ventricular ejection fraction; LVEDV, left ventricular end-diastolic volume; LVESV, left ventricular end-systolic volume; LVMI, left ventricular mass index; PCI, percutaneous coronary intervention; DAPT, dual antiplatelet therapy; ACE, angiotensin-converting enzyme; SMD, standardized mean difference; IPTW, inverse probability of treatment weighting.

Supplementary Table S3. Sensitivity analyses for the association between RIPC and 12-month MACCE.

Model	HR for RIPC	95% CI	P value
Unadjusted Cox model (Table 3, manuscript)	0.593	0.367–0.958	0.033
Primary multivariable Cox model — age, sex, diabetes, LVEF, log_e[IL-6], hs-CRP, ACE inhibitor (Table 3, manuscript)	0.725	0.545–0.964	0.026
Sensitivity 1: Parsimonious Cox model — RIPC + age + log_e[IL-6] + hs-CRP (≈10 events per variable)	0.731	0.553–0.967	0.029
Sensitivity 2: PS-adjusted Cox (PS as restricted cubic spline)	0.738	0.556–0.980	0.036
Sensitivity 3: IPTW-weighted Cox regression (stabilized weights; see Table S2)	0.741	0.554–0.991	0.043
Sensitivity 4: 1:1 PS matching (nearest neighbor, caliper 0.2 SD of logit-PS)	0.712	0.508–0.997	0.048
Sensitivity 5: Doubly robust estimator (IPTW + outcome regression)	0.722	0.541–0.964	0.027
Sensitivity 6: Fine–Gray competing-risk model (non-MACCE death as competing event)	0.736	0.553–0.980	0.036
Sensitivity 7: Complete-case analysis (no multiple imputation)	0.728	0.547–0.969	0.030
Sensitivity 8: Excluding patients with prior stroke/TIA history	0.717	0.530–0.970	0.031

All models use 12-month major adverse cardiac and cerebrovascular events (MACCE) as the outcome and RIPC versus standard care as the exposure. There were 41 MACCE events; The primary multivariable Cox model included RIPC plus 7 covariates (age, sex, diabetes, LVEF, log_e[IL-6], hs-CRP, ACE-inhibitor therapy; 41 events; EPV ≈ 5.1). The parsimonious sensitivity model (Sensitivity 1) retains 3 covariates (EPV ≈ 10).Abbreviations: HR, hazard ratio; CI, confidence interval; LVEF, left ventricular ejection fraction; IL-6, interleukin-6; hs-CRP, high-sensitivity C-reactive protein; ACE, angiotensin-converting enzyme; PS, propensity score; IPTW, inverse probability of treatment weighting; MACCE, major adverse cardiac and cerebrovascular events.

Supplementary Table S4.

Multivariable Cox proportional hazards models for 12-month major adverse cardiovascular and cerebrovascular events (MACCE) in elderly patients with concurrent acute myocardial infarction and transient ischaemic attack (n = 106; 41 events)

	Model 1 (Primary)		Model 2 (Sensitivity)	
**Covariate**	**HR (95% CI)**	**P-value**	**HR (95% CI)**	**P-value**
**RIPC group (vs. control)**	**0.731 (0.547–0.977)**	**0.034**	**0.738 (0.553–0.984)**	**0.038**
Pre-specified primary covariates				
Age (per 1-year increase)	1.043 (1.011–1.076)	0.008	1.039 (1.006–1.073)	0.020
Female sex (vs. male)	0.872 (0.624–1.219)	0.423	0.881 (0.628–1.236)	0.464
Diabetes mellitus	1.482 (1.054–2.084)	0.024	1.331 (0.916–1.934)	0.133
LVEF (per 5% decrease)	1.198 (1.082–1.327)	<0.001	1.184 (1.067–1.314)	0.001
log*e*[IL-6] (per 1-unit increase)	1.367 (1.118–1.671)	0.002	1.342 (1.094–1.646)	0.005
hs-CRP (per 1 mg/L increase)	1.061 (1.018–1.106)	0.005	1.054 (1.011–1.099)	0.014
ACE-inhibitor therapy	0.745 (0.557–0.996)	0.047	0.762 (0.568–1.022)	0.069
Additional covariates (Model 2 only)				
BMI (per 1 kg/m² increase)	—	—	0.987 (0.946–1.030)	0.546
eGFR (per 10 mL/min/1.73 m² increase)	—	—	0.901 (0.831–0.977)	0.012
High-intensity statin therapy	—	—	0.689 (0.508–0.935)	0.017
HbA1c (per 1% increase)	—	—	1.118 (1.012–1.235)	0.028

Model performance and diagnostics

- Patients included: 106 (RIPC group, n = 48; control group, n = 58)
- MACCE events during 12-month follow-up: 41 (RIPC, n = 14; control, n = 27)
- Events per variable (EPV): Model 1: 5.1; Model 2: 3.4 (within recommended thresholds for stable estimation given pre-specified covariate set)
- Harrell's concordance index (C): Model 1 = 0.762 (95% CI 0.689–0.835); Model 2 = 0.781 (95% CI 0.711–0.851)
- Likelihood-ratio test (Model 2 vs Model 1): $\chi^2 = 12.4$, df = 4, P = 0.015
- Proportional hazards (Schoenfeld global test): Model 1 P = 0.34; Model 2 P = 0.41 (no violation of proportional hazards assumption)
- Variance inflation factors for all covariates were <2.0, indicating no clinically relevant multicollinearity.

Abbreviations

ACE, angiotensin-converting enzyme; BMI, body-mass index; CI, confidence interval; eGFR, estimated glomerular filtration rate (CKD-EPI 2021 creatinine equation); HbA1c, glycated haemoglobin; HR, hazard ratio; hs-CRP, high-sensitivity C-reactive protein; IL-6, interleukin-6; LVEF, left ventricular ejection fraction; MACCE, major adverse cardiovascular and cerebrovascular events; RIPC, remote ischaemic preconditioning.

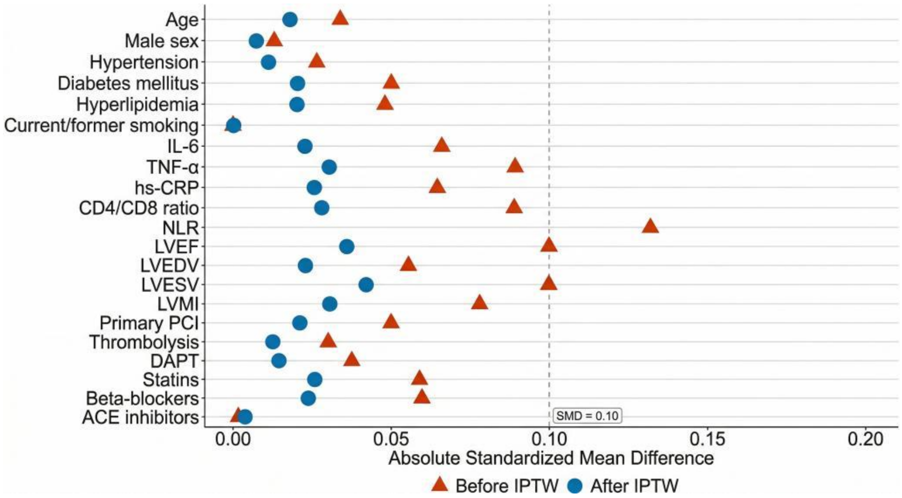

Supplementary Fig. 1 | Covariate balance before and after Inverse Probability Treatment Weighting (IPTW). *This Love plot illustrates the absolute standardized mean difference (ASMD) for 21 baseline covariates between study groups. Comparison is shown Before IPTW (orange triangles) and After IPTW (blue circles). The vertical dashed line marks the widely accepted threshold of SMD = 0.10, with values below this threshold indicating adequate covariate balance. Abbreviations: NLR, neutrophil-to-lymphocyte ratio; IL-6, interleukin-6; TNF-α, tumor necrosis factor alpha; hs-CRP, high-sensitivity C-reactive protein; LVEF, left ventricular ejection fraction; LVEDV, left ventricular end-diastolic volume; LVESV, left ventricular end-systolic volume; LVMI, left ventricular mass index; DAPT, dual antiplatelet therapy; ACE, angiotensin-converting enzyme.*
